# Antimitochondrial Antibody Negative Primary Biliary Cirrhosis in Japan: Utilization of Clinical Data When Patients Applied to Receive Public Financial Aid

**DOI:** 10.2188/jea.16.30

**Published:** 2005-12-20

**Authors:** Fumio Sakauchi, Mitsuru Mori, Mikio Zeniya, Gotaro Toda

**Affiliations:** 1 Department of Public Health, Sapporo Medical University School of Medicine.; 2 Division of Gastroenterology and Hepatology, Department of Internal Medicine, JIKEI University School of Medicine.; 3 Senpo Tokyo Takanawa Hospital.

**Keywords:** Liver Cirrhosis, Biliary, antimitochondrial antibody, Cross-Sectional Studies, complications

## Abstract

**BACKGROUND:**

We examined patients who showed laboratory and histological evidence of primary biliary cirrhosis (PBC) in the absence of antimitochondrial antibody (AMA) to elucidate the characteristics of AMA negative PBC.

**METHODS:**

From a total of 5,805 patients with symptomatic PBC, 2,419 cases (41.7%) were selected in the present study, who were diagnosed using the following criterion; chronic non-suppurative destructive cholangitis was histologically observed and laboratory data did not contradict PBC. The information collected from records included sex, age, symptoms, physical findings, and complicated autoimmune diseases. We then evaluated these data according to the positivity of AMA.

**RESULTS:**

Of the total subjects, 470 cases (19.4%) were found to be negative for AMA. The proportion of female patients was higher among the AMA negative group than among the AMA positive one. Pruritus was found less frequently among patients with AMA negative PBC than among those with AMA positive PBC. Levels of alkaline phosphatase, *γ*-glutamyl transpeptidase, and IgM were significantly lower among patients with AMA negative PBC than among those with AMA positive PBC. Complications such as Sjögren’s syndrome, rheumatoid arthritis, and scleroderma, including CREST syndrome, were found with significantly higher frequency among patients with AMA negative PBC than among those with AMA positive PBC.

**CONCLUSION:**

Considering serum level of IgM and frequencies of complicated autoimmune diseases, it is possible that Japanese patients with AMA negative PBC are consistent with the disease entity of autoimmune cholangitis reported in western countries.

Primary biliary cirrhosis (PBC) is a chronic cholestatic disorder characterized by progressive, nonsuppurative inflammation and destruction of small bile ducts. The presence of antimitochondrial antibody (AMA) in the sera is a very important finding for the diagnosis of PBC. However, it has been reported that some patients with laboratory and histological findings compatible with PBC do not have detectable AMA,^1^^,^^2^ and most information regarding AMA negative PBC is still limited to Europe and North America.

In Japan, symptomatic PBC was specified as one of “the intractable diseases” from 1990. Patients with symptomatic PBC who want to receive public financial aid from the Ministry of Health, Labour and Welfare have to sign agreements and write applications. They are then registered and can receive public financial aid. In 1999, the Ministry permitted the use of clinical data from the patients diagnosed with symptomatic PBC. Therefore, the data was available to the research committee of intractable hepatic diseases and the research committee on the epidemiology of intractable diseases. Using these data, we have already shown the clinical features of 5,805 prevalent cases of symptomatic PBC,^3^ whose conditions met one of the three criteria outlined by the previous reports in Japan;^4^^-^^6^ (1) chronic non-suppurative destructive cholangitis is histologically observed and laboratory data do not contradict PBC; (2) AMA is positive, and chronic non-suppurative destructive cholangitis is not histologically observed but histological findings are compatible with PBC; and (3) histological examination is not performed, but AMA is positive and clinical findings and course indicate PBC.

In the present study, we tried to elucidate the characteristics of AMA negative PBC in Japan using the clinical data when they applied to receive the public financial aid.

## METHODS

In the fiscal year 1999, 9,761 prevalent cases with symptomatic PBC were registered; they were about 81% of 12,000 patients estimated in Japan.^7^ We used the clinical data of 6,305 patients being that not all prefectures provided the data, and chose the cases of 5,805 patients whose clinical data were written and collected between 1999 and 2000; the data from residual cases were not written during this time, for example written in 1998 or before. From these 5,805 patients, 2,419 cases (41.7%) who were diagnosed according to the above-mentioned criterion,^1^ i.e. chronic non-suppurative destructive cholangitis was histologically observed and laboratory data did not contradict PBC, were selected in the present study. After then the following information of each patient was collected from the records; sex, age, symptoms and physical findings, complicated autoimmune diseases, laboratory data including serum levels of bilirubin, alkaline phosphatase (ALP), *γ*-glutamyl transpeptidase (*γ*-GTP), total cholesterol, IgM, and frequencies of positivity of AMA. We evaluated symptoms and physical findings, laboratory data, and complicated autoimmune diseases according to the positivity of AMA. In the present study, the frequencies of items in the clinical data were analyzed, excluding “unclear” or blank spaces.

Statistical analysis was performed using SPSS^®^ version 10.0 (SPSS Inc.). The chi-square test was used for comparing the proportions of two groups, and the Mann-Whitney test was used to evaluate differences in clinical variables. P < 0.05 was considered significant.

## RESULTS

### Sex and Age

Of the total subjects, 470 of the 2,419 cases (19.4%) were found to be negative for AMA. Table 1 presents male-to-female ratios (men/women) and age distributions according to positivity of AMA. The male-to-female ratio was 0.14 among the AMA positive group, and was 0.09 among the AMA negative one, therefore, the proportion of female cases was higher among the AMA negative group than among the AMA positive one (P=0.01). The median of age was 58 years among AMA positive cases, and was 59 years among AMA negative cases with no significant difference (P=0.53).

**Table 1.** Frequencies of patients with primary biliary cirrhosis according to positity of antimitochondial antibody (AMA).

		All subjects	AMA (+)	AMA (−)
Male/female ratio		0.13 (278 /2,141)	0.14 (241/1,708)	0.09 (37/433)*
Age (year): Median (interquartile range)		58 (51 - 66)	58 (50 - 66)	59 (52 - 65.5)^†^

Age group (year)	-49	21.5% (519)	22.1% (430)	18.9% ( 89)
	50-59	32.1% (777)	32.0% (623)	32.8% (154)
	60-69	33.7% (815)	32.8% (640)	37.2% (175)
	70+	12.7% (308)	13.1% (256)	11.1% ( 52)
	Total	100% (n=2,419)	100% (n=1,949)	100% (n=470)

* : *χ*^2^ test for AMA(+) vs. AMA(-), P=0.01

† : Mann-Whitney test for AMA(+) vs. AMA(-), P=0.53

### Symptoms and Physical Findings

Pruritus was found significantly less frequent in patients with AMA negative PBC than among those with AMA positive PBC (P=0.03) (Table 2). Splenomegaly was also found less frequent in patients with AMA negative PBC, but not significant, while frequencies of jaundice, xanthomas, and esophageal varices were almost the same between AMA negative and positive patients. We also conducted Mantel-Haenszel test to control the male-to-female ratio, but significant differences between the two groups did not change.

**Table 2.** Symptoms and physical findings among patients with primary biliary cirrhosis according to positivity of antimitochondrial antibody (AMA).

	All subjects	AMA (+)	AMA (−)	P value*	P value^†^
Pruritus	53.9% (1,274/2,362)	55.1% (1,048/1,903)	49.2% (226/459)	0.03	0.03
Jaundice	10.5% ( 250/2,387)	10.8% ( 208/1,927)	9.1% ( 42/460)	0.34	0.37
Xanthomas	6.5% ( 151/2,311)	6.5% ( 121/1,864)	6.7% ( 30/447)	0.95	0.92
Splenomegaly	37.4% ( 876/2,344)	38.2% ( 722/1,899)	33.8% (154/455)	0.09	0.09
Esophageal varices	16.5% ( 385/2,171)	16.8% ( 294/1,755)	15.4% ( 64/416)	0.55	0.50

Denominators are not equal because frequencies of items were analyzed excluding “unclear” or blank spaces.

* : *χ*^2^ test for AMA (+) vs. AMA (−)

† : Mantel-Haenszel test for AMA(+) vs. AMA(−) to control male/female ratio

### Laboratory Data

Key laboratory data are summarized in Table 3. Levels of ALP, *γ*-GTP, and IgM were significantly lower among patients with AMA negative PBC than among those with AMA positive PBC.

**Table 3.** Laboratory findings among patients with primary biliary cirrhosis according to positivity of antimitochondrial antibody (AMA).

	All subjects		AMA(+)		AMA(−)		P value*
		
	Median	Interquartile range	Median	Interquartile range	Median	Interquartile range	
Total bilirubin (mg/dL)	0.6 (n=2,404)	0.5 -0.9	0.6 (n=1,843)	0.5 -0.9	0.6 (n=437)	0.5 -0.9	0.06
ALP (IU/L)	355 (n=2,530)	243-566	364 (n=1,938)	249-575	326 (n=465)	221-522	0.01
*γ*-GTP (IU/L)	84 (n=2,521)	37-194	91 (n=1,929)	40-203	65 (n=465)	29-165	<0.001
Total cholesterol (mg/dL)	207 (n=2,417)	178-235	207 (n=1,867)	178-235	208 (n=443)	176-235	0.65
IgM (mg/dL)	343 (n=2,060)	209-552	376 (n=1,603)	230-594	239 (n=396)	154-395	<0.001

*: Mann-Whitney test for AMA(+) vs. AMA(−)

### Complicated Autoimmune Diseases

Complications such as Sjögren’s syndrome, rheumatoid arthritis, chronic thyroiditis, and scleroderma, including CREST syndrome (calcinosis, Raynaud’s phenomenon, esophageal dysmotility, sclerodactyly, and telangiectases) were found in 20.5%, 9.7%, 6.0%, and 4.0% of the patients with AMA negative PBC, respectively (Table 4). These complicated diseases, excluding chronic thyroiditis, were found with significantly higher frequency in patients with AMA negative PBC than among those with AMA positive PBC. We also conducted Mantel-Haenszel test to control the male-to-female ratio, but significant differences between the two groups did not change.

**Table 4.** Complicated autoimmune diseases among patients with primary biliary cirrhosis according to positivity of antimitochondrial antibody (AMA).

Autoimmune diseases	All subjects	AMA (+)	AMA (−)	P value*	P value^†^
Sjögren’s syndrome	16.6% ( 356/2,141)	15.7% ( 271/1,726 )	20.5% ( 85/415 )	0.02	0.04
Rheumatoid arthritis	7.0% ( 161/2,294 )	6.4% ( 118/1,850 )	9.7% ( 43/444 )	0.02	0.04
Chronic thyroiditis	4.3% ( 103/2,419 )	3.8% ( 75/1,949 )	6.0% ( 28/470 )	0.06	0.054
Scleroderma	2.4% ( 59/2,419 )	2.1% ( 40/1,949 )	4.0% ( 19/470 )	0.02	0.03

Denominators are not equal because frequencies of items were analyzed excluding “unclear” or blank spaces.

* : *χ*^2^ test for AMA (+) vs. AMA (−)

† : Mantel-Haenszel test for AMA(+) vs. AMA(−) to control male/female ratio

## DISCUSSION

AMA is the serologic hallmark of PBC. However, a small number of patients with PBC lack AMA, as such these patients are often referred as AMA negative PBC. When patients who have cholestatic disorders are found, it is necessary to differentiate PBC from other diseases causing cholestasis, such as primary sclerosing cholangitis.^8^ In the present study, therefore, cases that had been diagnosed by histological confirmation were selected.

Our patients with AMA negative PBC presented some revealing features; (1) The proportion of female cases was higher among the AMA negative group than among the AMA positive one. (2) Pruritus was found less frequently among patients with AMA negative PBC than among those with AMA positive PBC. (3) Levels of ALP, *γ*-GTP, and IgM were lower among patients with AMA negative PBC. And (4) Complicated autoimmune diseases were found more frequently among patients with AMA negative PBC. It may be one explanation to the frequencies of pruritus and complicated autoimmune diseases in two groups that the AMA negative group included more women than the AMA positive one. It could be that hormonal differences between the sexes may exert modulating factors that predispose each sex toward a particular immune response.^9^^,^^10^ However, we cannot immediately conclude that these statistical differences are truly meaningful, because we dealt a large number of cases with PBC in the present study. Fore example, difference in percentage of pruritus was 6% and not so large. Further studies of the pathophysiological mechanism of PBS will need to be conducted in order to explain these differences comprehensively.

Michieletti et al.^11^ and Lacerda et al.^12^ reported that the AMA negative group had significantly lower serum IgM than the AMA positive controls, but the level of ALP showed no significant difference between the AMA negative and positive groups. Regarding the level of IgM, our result was consistent with these two reports, but our AMA negative group had significantly lower levels of ALP, and *γ*-GTP compared with the AMA positive group. One possible explanation may be the number of cases examined; In the previous studies, 17 and 35 cases with AMA negative PBC were examined respectively, whereas we examined 470 AMA negative PBC cases. Although Michieletti et al.^11^ and Lacerda et al.^12^ showed that the AMA negative group had lower levels of ALP compared with the AMA positive group, these were not statistically significant. Sánchez-Pobre et al. also reported lower serum levels of ALP and IgM among AMA negative PBC patients.^13^

Ben-Ari et al. described four AMA negative patients with overlapping features of PBC and autoimmune chronic active hepatitis,^14^ and it was considered that such a subgroup might be termed autoimmune cholangiopathy;^15^ sometimes AMA tests by immunofluorescence technique (IFT) were negative but other autoantibodies such as antinuclear antibody were positive.^16^^,^^17^ In addition, ‘autoimmune cholangitis’ was proposed in some reports.^11^^,^^12^^,^^18^^-^^21^ Michieletti et al. believed that AMA negative patients have a form of autoimmune cholangitis distinct from that seen in AMA positive PBC and other autoimmune liver diseases.^11^ Lacerda et al. presented AMA negative PBC cases having other atutoantibodies, such as antinuclear antibody and antismooth muscle antibody, despite the chronic non-suppurative destructive cholangitis in liver biopsy specimens.^12^ On the other hand, the belief that PBC and autoimmune cholangitis are part of the same spectrum of immunologically mediated disease has risen recently.^22^ This spectrum would include classic AMA positive PBC as well as the syndrome with the absence of AMA in the serum. Perhaps a similar pathogenetic mechanism may underlie these diseases.^22^^-^^24^ It was also suggested that a part of AMA negative PBC might rapidly progress to liver cirrhosis within a short time.^13^ Future research is required to clarify the relationship between AMA positive and negative PBC.

Serum from patients suspected of having PBC is usually tested by IFT or enzyme linked immunosorbent assay (ELISA) for the detection of AMA. Most patients with PBC have AMA when serum samples are tested by IFT, but it was suggested that 1-16% of PBC patients do not have AMA when tested by IFT.^11^^,^^21^^,^^22^^,^^25^ Moreover, the prevalence of positivity of AMA by ELISA method is reported to be 92-93% among patients with PBC.^16^ It has been suggested that patients with AMA negative results may be shown to have AMA by more sophisticated detection techniques.^25^^,^^26^ For detecting AMA, more sensitive assays, such as immunoblotting, have been developed, and this method is expected to prove AMA positivity for most patients with AMA negative PBC by IFT or ELISA.^26^

The present study has several limitations. Firstly, we cannot completely deny that our AMA negative cases obtained by IFT or ELISA turn into AMA positive ones by other AMA detection methods such as immunoblotting. This is a critical problem that needs to be resolved, but it may be difficult to generalize immunoblotting methods for detecting AMA because of the complex nature of their techniques and expenditure. Secondly, other clinical findings including level of IgG, autoimmune antibodies such antinuclear antibody and antismooth muscle antibody were not available, because our patients’ protocols had only the information essential for their receiving public financial aid. Additionally, we could not get adequate information about treatment and use of drugs, such as ursodeoxycholic acid. Nevertheless, to our knowledge, the present study has reported the largest number of AMA negative PBC in Japan, and we consider that we could present an outline on AMA negative PBC.

In conclusion, considering serum level of IgM and frequencies of complicated autoimmune diseases, it is possible that Japanese patients with AMA negative PBC are consistent with the disease entity of autoimmune cholangitis reported in western countries.
